# Impact of single freeze-thaw cycles on human serum proteins: Implications for mass spectrometry biomarker validation

**DOI:** 10.1016/j.isci.2026.115934

**Published:** 2026-04-28

**Authors:** Thorben Sauer, Martina Oberländer, Regina Maushagen, Helene Radloff, Ruediger Braun, Kim Honselmann, Ulrich Wellner, Jens Habermann, Claudia Benecke, Tobias Keck, Silke Szymczak, Timo Gemoll

**Affiliations:** 1 Section for Translational Surgical Oncology and Biobanking, Department of Surgery, University of Lübeck and University Hospital Schleswig-Holstein, Campus Lübeck, 23538 Lübeck, Germany; 2 Interdisciplinary Center for Biobanking-Lübeck (ICB-L), University Hospital Schleswig-Holstein, Campus Lübeck, 23538 Lübeck, Germany; 3 Department of Surgery, University Hospital Schleswig-Holstein, Campus Lübeck, 23538 Lübeck, Germany; 4 Institute of Medical Biometry and Statistics, University of Lübeck, 23562 Lübeck, Germany

**Keywords:** Oncology, Proteomics

## Abstract

Validation of biomarkers for clinical diagnostics requires high-quality samples and standardized protocols. This study investigated the effect of a single freeze-and-thaw (FT) cycle on the quality of liquid nitrogen-stored clinical serum samples. Using mass spectrometry, serum protein abundance was analyzed in a human test cohort of 99 samples and a validation cohort 109 samples. After preprocessing, 213 proteins were analyzed in the test cohort and 248 in the validation cohort. Thirty proteins changed significantly in their measurability after freeze-thaw in the test cohort, and 11 were confirmed in the validation cohort. Among them, CP and IGHV6-1 showed potential for distinguishing malignant from benign pancreatic disease. No association was found between protein intensity and storage duration. Overall, the findings show that freeze-thaw cycles are an important pre-analytical factor that should be controlled when translating protein biomarkers into clinical practice.

## Highlights

• A single freeze-thaw cycle alters human serum protein abundance patterns in DIA-MS
• 11 serum proteins are validated as FT-sensitive across two independent DIA-MS cohorts
• FT-sensitive proteins are mostly hydrophobic and thermodynamically unstable
• Freeze-thaw is a pre-analytical factor that may impair biomarker clinical translation

## Graphical abstract

**Figure:**
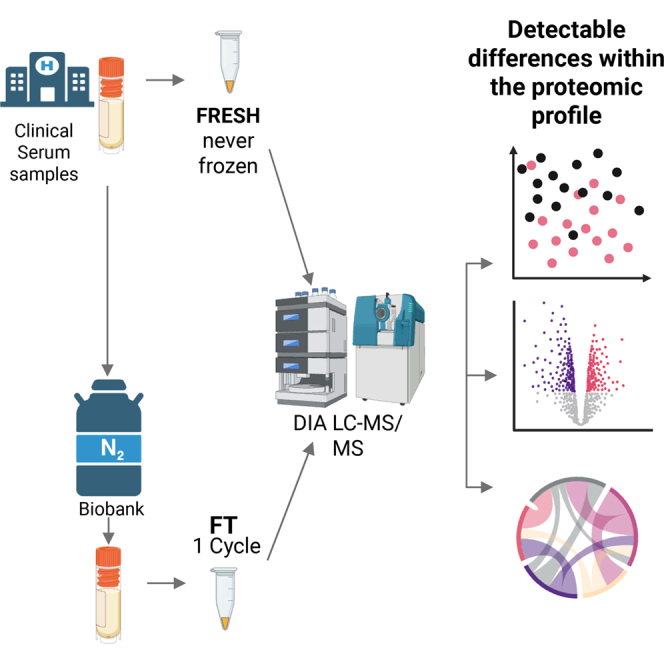

## Introduction

Cancer is one of the leading global health challenges, responsible for nearly 10 million deaths in 2020 alone, and is projected to become the leading cause of mortality by 2060.^1^ The rising incidence of various cancers—driven by factors such as lifestyle choices, environmental exposures, genetic predisposition, and an aging population—highlights an urgent need for effective diagnostic and therapeutic strategies.

Among recent advances, liquid biopsies and protein-based biomarkers have emerged as promising approaches for non-invasive detection and real-time monitoring of tumor dynamics.^2^^,^^3^ Extensive basic research has led to the identification of numerous biomarker candidates, raising hopes for improved clinical decision-making. Biobanks, characterized by stringent regulatory frameworks and detailed sample collection, provide high-quality repositories of patient biospecimens and associated clinical metadata. These resources support large-scale retrospective studies that accelerate biomarker discovery and validation.

The translation of candidate biomarkers into clinically relevant diagnostic tools presents a persistent challenge, despite numerous discoveries. Most candidates fail to demonstrate sufficient sensitivity and specificity across diverse populations, resulting in a persistent gap between laboratory discoveries and clinical applications. Key limitations include deficiencies in study design, execution, and technical considerations during the early stages of biomarker research.^4^ Critically, pre-analytical factors impacting sample quality—such as storage conditions and freeze-thaw (FT) cycles—are frequently underestimated. While bio-banked samples typically subjected to standardized handling and storage protocols, clinical samples may be analyzed fresh, without exposure to FT cycles that could introduce significant biases. Research indicates that inadequate sample handling can lead to degradation of RNA, DNA, metabolites, and proteins,^5^^,^^6^ with FT cycles specifically causing denaturation, aggregation, and degradation of serum and plasma proteins. These effects have the potential to alter both the abundance and detectability of candidate biomarkers. However, current research on the impact of FT cycles remains limited in scope: existing studies often examine only a small subset of proteins, and direct comparisons between fresh samples and those undergoing a single FT cycle are notably absent.^7^^,^^8^^,^^9^^,^^10^ Such analyte discrepancies may, therefore, contribute to the lack of reproducibility and poor clinical translation of promising biomarkers.

In light of these challenges, the present study was designed to comprehensively evaluate the FT cycle as a pre-analytical factor affecting global proteome profiles of cancer patient serum samples. Using data-independent acquisition mass spectrometry (DIA-MS), paired analyses of fresh and FT serum samples were performed to detect distinct protein abundance patterns under each condition. This study aims to address the existing knowledge gap regarding pre-analytical variability, with the objective of improving biomarker reliability and its translation into clinical practice.

## Results

### Study workflow and cohort description

**Figure 1:**
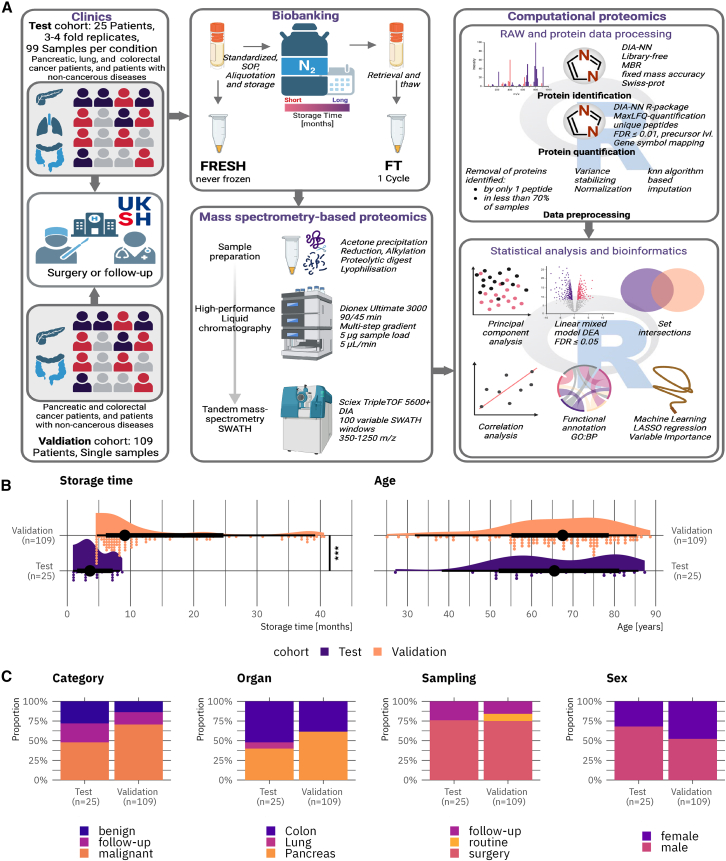
Study workflow and patient cohort meta data visualization (A) Study workflow description. Blood samples from two independent cohorts were collected in the clinic. Serum was derived from blood samples in the biobank and sample aliquots were stored in liquid nitrogen. Fresh aliquots (never frozen) were immediately processed and prepared for mass-spectrometry-based proteomics. FT samples (one freeze-and-thaw cycle) were extracted after a certain storage time and identically processed. Fresh and FT samples were analyzed with LC-MS/MS in data-independent acquisition mode. Acquired RAW data were processed in DIA-NN and the protein data were stringently preprocessed in R. Statistical and bioinformatics analyses were carried out in R and include clustering analyses, differential abundance analyses, correlation analyses, and functional annotations. A sub-cohort was used for a biomarker discovery simulation using machine learning. (B) Raincloud plots visualizing the distribution of storage time (months) and patient age (years) across both study cohorts Asterisks indicate significance in the Wilcoxon rank-sum test: ∗∗∗ = *p* value ≤0.001. (C) Stacked bar plots representing metadata variables across both study cohorts.

This study investigated the pre-analytical effects of a single FT cycle on the serum proteome, specifically within the context of cancer biomarker research (Figure 1A). A paired comparison was conducted, analyzing serum samples in their fresh state (never frozen) and after undergoing a single FT cycle following storage in liquid nitrogen for varying durations. The study employed a test cohort of 99 fresh-FT sample pairs from 25 patients and a validation cohort of 109 fresh-FT sample pairs derived from 109 patients. The samples were subjected to mass spectrometry-based proteomics in data-independent acquisition. Computational proteomics, incorporating rigorous data pre-processing of the RAW and protein data, statistical analysis, and bioinformatics, was subsequently performed. This analysis aimed to evaluate protein profiles of fresh and FT serum samples and to characterize the impact of an FT cycle on the proteome. The descriptive statistical analysis results are presented in Table 1, while Figures 1B and 1C illustrates the variable distribution across both cohorts. The median storage duration for the test cohort was 3.6 months (inter-quartile-range 2.8; 7.1, standard deviation 2.5), while the validation cohort demonstrated a median storage time of 9.1 months (inter-quartile-range 6.7; 19.9, standard deviation 11.0). The diseased organ distribution was different between the cohorts. Specifically, the validation cohort comprises a higher proportion of patients with colon diseases, while pancreatic diseases were predominant within the validation cohort.

**Table 1:** Comparison of study cohorts based on clinical and sample characteristics

		Test (*n* = 25)	Validation (*n* = 109)
**Clinical characteristics**			

Age (years)	Min/max	27.1/87.3	25.1/88.7
	Median (first quartile; third quartile)	65.5 [56.2;79.8]	67.5 [57.8;75.3]
	Mean (SD)	66.3 (15.0)	65.4 (14.0)
Sex	Female	8 (32.0%)	52 (47.7%)
	Male	17 (68.0%)	57 (52.3%)
Organ	Colon	13 (52.0%)	42 (38.5%)
	Lung	2 (8.0%)	0 (0%)
	Pancreas	10 (40.0%)	67 (61.5%)
Diagnosis	Colon adenoma	0 (0%)	2 (1.8%)
	Colorectal cancer	12 (48.0%)	39 (35.8%)
	Diverticulitis	0 (0%)	1 (0.9%)
	Duodenal polyps	1 (4.0%)	0 (0%)
	Intraductal papillary mucinous neoplasm	3 (12.0%)	6 (5.5%)
	IPMN based pancreatic cancer	0 (0%)	4 (3.7%)
	Lung cancer	2 (8.0%)	0 (0%)
	Pancreatic adenoma	0 (0%)	1 (0.9%)
	Pancreatic cancer	4 (16.0%)	38 (34.9%)
	Pancreatic cyst	0 (0%)	3 (2.8%)
	Pancreatic neuroendocrine tumor	0 (0%)	10 (9.2%)
	Pancreatitis	3 (12.0%)	4 (3.7%)
	Unknown pancreatic neoplasia	0 (0%)	1 (0.9%)
Category	Malignant	12 (48.0%)	77 (70.6%)
	Benign	7 (28.0%)	15 (13.8%)
	Follow-up	6 (24.0%)	17 (15.6%)

**Sample characteristics**			

Sampling	Follow-up	6 (24.0%)	17 (15.7%)
	Routine	0 (0%)	10 (9.3%)
	Surgery	19 (76.0%)	81 (75.0%)
	NA	0	1
Storage time (months)	Min/max	0.9/8.7	4.5/40.6
	Median (first quartile; third quartile)	3.6 [2.8;7.1]	9.1 [6.7;19.9]
	Mean (SD)	4.2 (2.5)	14.2 (11.0)

### Proteomics data quality and comparability

**Figure 2:**
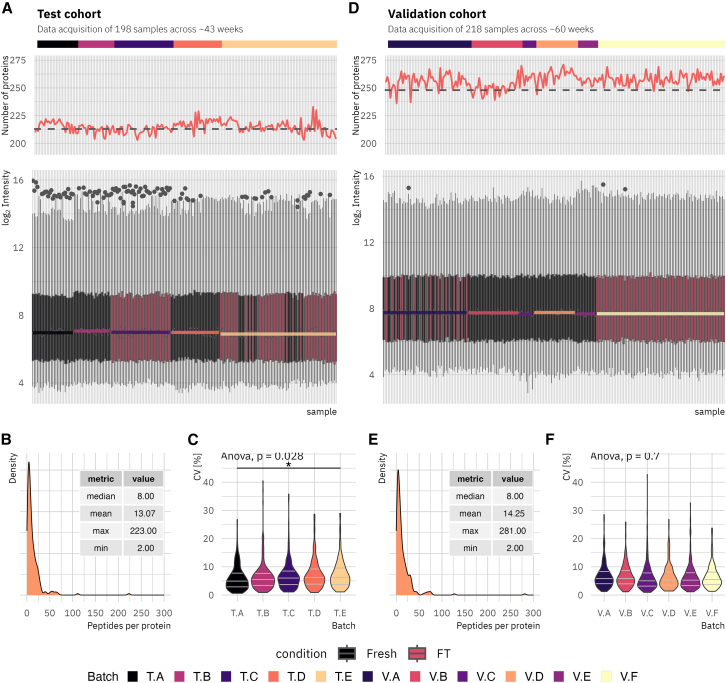
Proteomics data quality control for test and validation cohort data The test cohort data (*n* = 99) were acquired in five batches over approximately 43 weeks. The validation cohort (*n* = 109) samples were measured in six batches over approximately 60 weeks. (A and D) Visualization of the normalized log_2_ intensity for all samples in chronological order as boxplots, the number of proteins identified per sample before filtering as line plot (dashed line indicates the number of proteins in the final, pre-processed dataset), as well as the corresponding acquisition batch per sample. Colored lines in boxplots represent the average median of the corresponding batch. (B and E) Density plots visualizing the distribution of identified peptides per protein, including descriptive metrics as table. (C and F) Violin plots visualizing the distribution of the proteins CV per analysis batch. Gray lines within violins indicate quantiles. ANOVA was used to evaluate global CV significance. Asterisks indicate significance in student’s *t* test: ∗ = adj. *p* value ≤0.05.

Serum proteomic profiles were generated for both study cohorts using a library-free data analysis approach. The test study resulted in a proteomic dataset of 198 samples, acquired across five batches over a period of approximately 43 weeks. The validation cohort data encompassed 218 samples, distributed across six batches over approximately 60 weeks. Protein identification rates remained consistent for both proteomic datasets throughout the acquisition period and across all acquisition batches. The test cohort analysis identified between 203 and 230 unique proteins (mean 214.7, standard deviation 5.08), while the validation cohort analysis identified between 236 and 271 unique proteins (mean 256.5, standard deviation 6.43, Figures 2A and 2D). Following pre-processing, the datasets contain relative abundance data for 213 unique proteins in the test cohort and 248 unique proteins in the validation cohort. The number of identified peptides per protein was consistent across both cohorts, with a mean of eight peptides per protein (Figures 2B and 2E). A statistically significant difference in mean coefficient of variation (CV) of measured protein intensity between batches was identified exclusively within the test cohort (Anova *p*-value = 0.028), while no such difference was evident in the validation cohort (*p* value = 0.7) (Figures 2C and 2F). Pairwise comparisons between test study batches revealed a significant difference in the mean CV for T.A versus T.D (student’s *t* test adj.*p* ≤ 0.05). However, these results suggest a high degree of reproducibility and inter-batch comparability; therefore, no batch correction was applied to the datasets.

### FT cycle as pre-analytical impact factor

**Figure 3:**
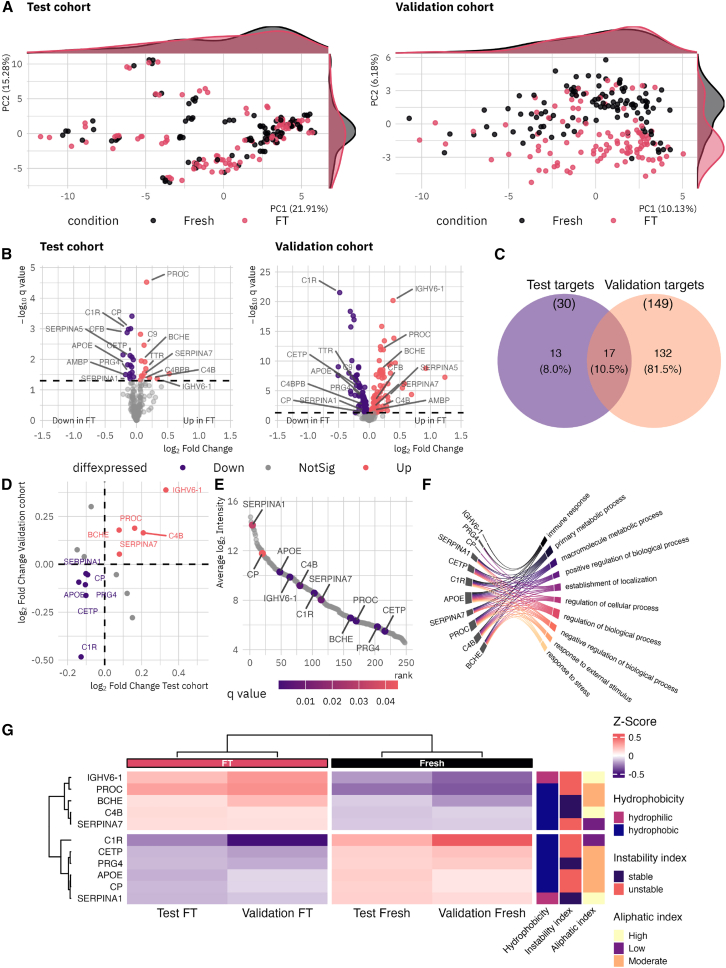
Proteomic profiling results for the test (*n* = 99) and validation (*n* = 109) cohorts (A) Principal component analysis plots for the test and validation cohorts. While the clustering of the test cohort is relatively indistinct, the clustering of the validation cohort shows clear tendencies. Adjacent density plots show the sample group overlaps by showing the kernel densities. Percentages of total variance explained by each component are shown in parentheses. (B) Volcano plots visualize the results of differential intensity analysis using LMMs. Significance is considered at *q* values ≤0.05. While 30 proteins were found to be differentially abundant in the test cohort, 149 proteins showed significant differences in abundance in the validation cohort. Protein names highlight proteins identified as differentially abundant in both cohorts. (C) Venn diagram visualizing the total overlap of significant proteins identified in the test and validation studies. (D) Scatterplot visualizing the fold change of each protein in test and validation cohort. (E) Protein rank plot from highest to lowest abundant proteins (calculated from the validation cohort), illustrating the dynamic range of target proteins. (F) Chord plot visualizing the top ten gene ontology mappings for the biological process subontology, selected by gene ratio. (G) Heatmap illustrating average *Z* score transformed protein intensities for target proteins, calculated by cohort and condition (fresh and FT). Heatmap annotations illustrate important physiochemical properties: Kyte and Doolittle hydrophobicity scale (discretized: >0 ≙ hydrophobic, <0 ≙ hydrophilic), Guruprasad instability index (discretized: >40 ≙ unstable, <40 ≙ stable), and Ikai aliphatic index (discretized: <70 ≙ low, 70–90 ≙ moderate, >90 ≙ high).

A principal component analysis was performed on both proteomic datasets to assess global disparities in proteomic profiles. The analysis revealed a relatively ambiguous clustering of FT and fresh samples of the test cohort, largely influenced by the high similarity of sample replicates (Figure 3A). In contrast, the validation cohort exhibited a distinct clustering trend, particularly captured by PC2 (6.1% total explained variance).

To identify proteins exhibiting significant intensity variations in FT samples, linear mixed models were applied to the proteomic profiles of both the test and validation cohorts. The analysis revealed statistically significant differential intensity (q-value ≤0.05) for 30 proteins when comparing paired fresh and FT samples of the test cohort (Figure 3B). Specifically, 17 proteins (57%) demonstrated increased intensity, while 13 (43%) exhibited decreased intensity in FT samples (detailed results for the test cohort are available in Table S2). Overall, 14% of the identified serum proteins demonstrated a statistically significant difference in measurable intensity following one FT cycle. The validation study further corroborated these findings, with 149 proteins (60%) demonstrating a statistically significant alteration in measured intensity following one FT cycle due to storage in liquid nitrogen (Figure 3B). Notably, the proportion of proteins exhibiting increased or decreased intensities in FT samples was consistent with the test study. Specifically, 70 (47%) displayed an increased fold change, while 79 proteins (53%) showed decreased intensities (detailed results for the validation cohort are available in Table S3). A Venn diagram, depicted in Figure 2C, illustrates the intersection of differentially expressed proteins. Among the 162 unique proteins identified as FT-sensitive, 17 were observed in both the test and validation cohorts. Of these 17 proteins, 11 proteins exhibited consistent directional changes (either decreased or increased intensity) across both cohorts (Figure 3D). These 11 proteins were designated as validated FT-sensitive proteins and spanned approximately eight orders of magnitude (Figure 3E). However, the Pax.Db for human serum was utilized for the binning of the listed proteins by abundance percentiles. The mapping of these abundance classes to the dataset under investigation revealed that the majority of the proteins identified in this study are high abundant proteins (87% of mappable protein identifiers), while 12% are mid, and less than 1% are low abundant proteins. The abundance stratification of each identified protein is documented in Table S4. Functional annotation by mapping the gene symbols to the subontology “biological process” of the gene ontology (GO) database revealed involvement in the immune response, metabolic processes, cellular and biological processes, the establishment of localization, and the response to stress (Figure 3F). Detailed GO:BP mapping results are available in Table S5.

A data mining approach was applied to assess the physiochemical attributes of the target proteins.^11^ In this context, three characteristics were identified as particularly relevant: hydrophobicity index, instability index, and aliphatic index. All indices were calculated based on the primary amino acid sequence of each protein. Figure 3G depicts the protein annotation results, including the mean relative protein intensity by condition and cohort. Of the 11 proteins analyzed, only two showed hydrophilic characteristics (index >0 ≙ hydrophobic, <0 ≙ hydrophilic). Seven of the 11 presented an instability index >40, indicating instability (index >40 ≙ unstable, <40 ≙ stable). Two proteins demonstrated a low aliphatic index, six were moderate, and three had a high aliphatic index (index <70 ≙ low, 70–90 ≙ moderate, >90 ≙ high). Each characteristic phenotype was represented by proteins with either increased or decreased intensity following the FT cycle. The target protein data, along with the physiochemical annotation results, are available in Table S6.

To evaluate the impact of the storage time in liquid nitrogen on quantifiable protein intensity within FT samples, a Pearson correlation analysis was performed using all FT samples from the validation cohort. The results indicated no significant correlation between storage time and protein intensity. The correlation analysis results are visualized in Figure S1. Complement component 1r (C1R) exhibited the highest net Pearson correlation coefficient (R = 0.22, *p* value = 0.019). Furthermore, no strong correlation was identified between the storage time and patient age, with Cholinesterase showing the highest net R of −0.36 (*p* value = 0.0001) (see Figure S1).

In summary, 28.2% of the identified proteins demonstrated consistent FT-stability in both the test and validation dataset. Further analysis revealed that 4.4% of proteins exhibited validated FT-sensitivity, while 2.4% displayed FT-sensitivity in both datasets but with discordant directionality. A further 50.0% showed FT-sensitivity in only one dataset, and 14.9% of proteins were exclusively identified in the validation set (8.5% exhibiting sensitivity, 6.4% exhibiting stability). A comprehensive summary of the stability status for each identified protein is available in Table S4. These findings suggest that a single FT cycle is sufficient to induce a sustainable effect on serum samples, which is quantifiable through both clustering and differential expression analyses. The duration of storage does not appear to influence the levels of FT-sensitive proteins.

## FT-sensitive proteins are potential biomarker candidates

**Figure 4:**
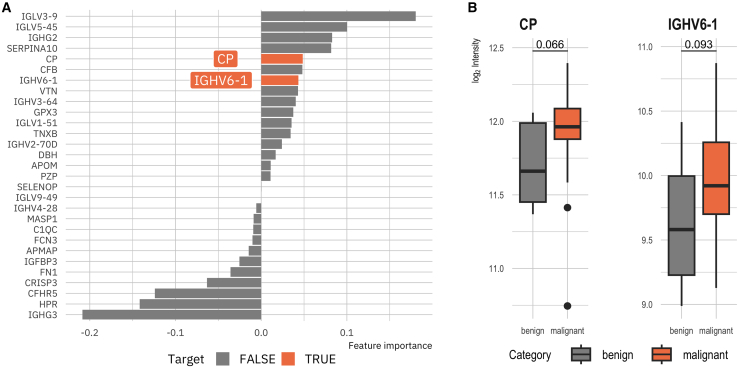
Biomarker candidate identification for classification into malignant and benign pancreatic diseases A data subset from 37 fresh validation cohort serum samples was used (27 malignant, ten benign). (A) LASSO regression feature importance (non-zero coefficients) results used for feature selection. Two target proteins were among the 29 features with an estimate ≠ 0. (B) Boxplots visualizing the measured log_2_ intensity of CP and IGHV6-1. *p* values derived with student’s *t* test.

The identification of FT-sensitive proteins as potential diagnostic biomarker candidates presents a challenge for the translation of findings from academic research, where FT samples are common, to clinical applications, which typically utilize fresh samples. Consequently, fresh serum proteomics data from a sub-cohort of 37 patients within the validation cohort were analyzed. This sub-cohort comprised patients with malignant pancreatic diseases (pancreatic ductal adenocarcinomas/PDAC, *n* = 27) and benign pancreatic lesions (*n* = 10). Details regarding sample inclusion are provided in Table S1. LASSO regression analysis identified 29 proteins as relevant for this classification problem, with non-zero estimated coefficients. The model achieved an accuracy of 0.62 and an area under the receiver operator characteristics curve of 0.67. Notably, two of these proteins were also identified as FT-sensitive: IGHV6-1 (immunoglobulin heavy variable 6-1) and CP (Ceruloplasmin) (Figure 4A). Both proteins exhibited an increased median log_2_ intensity in malignant samples compared to benign samples (Figure 4B). These findings indicate that FT-sensitive proteins may be identified as proteomic diagnostic biomarkers in serum samples highlighting the significance of FT-cycle-mediated factors in translational research.

## Discussion

The translation of biomarker candidates from basic research to clinical application remains a substantial challenge in modern medicine. While rapid advancements in omics technologies have facilitated the identification of numerous promising biomarker candidates for novel diagnostics and therapeutics, the translation process is hindered by obstacles such as a lack of standardized protocols for sample collection and processing, as well as insufficient quality control measures.^4^^,^^12^^,^^13^ These factors can result in the identification of biomarkers that lack the necessary sensitivity and specificity for clinical use. This study provides a fit-for-purpose evaluation of how a storage-related FT cycle affects relative peptide- and protein-level readouts measured by untargeted mass spectrometry. Mass spectrometry’s ability to process fresh samples directly to stable tryptic peptides minimizes additional pre-analytical variability compared to intact-protein assays. Over 200 fresh and FT serum sample pairs were examined. The cohorts in this study were not matched for disease type, stage, sex, or age, intentionally reflecting the heterogeneity typically encountered in real-world biobank collections. This diversity supports the generalizability of the observed freeze-thaw effects across different clinical backgrounds, although potential disease-related confounding cannot be fully excluded.

Mass spectrometry analysis yielded abundance data for 213 proteins in the test cohort and 248 proteins in the validation cohort, encompassing mid-to high-abundance serum proteins (Table S4). Although advanced proteomic techniques employing state-of-the-art mass spectrometers facilitate deeper proteomic coverage and the quantification of low-abundance proteins,^14^^,^^15^^,^^16^ we hypothesize that the observed proteomic alterations following a single FT cycle may also apply to both mid- and low-abundance proteins.

The proteomic data quality was rigorously evaluated throughout the duration of the study (43 weeks for the test and 60 weeks for the validation cohort). Despite certain limitations in the study design, including potential confounding of sample types within specific batches, the analysis revealed no significant batch effects related to sample processing order, signal intensities, storage duration, or protein identifications. These findings demonstrate the robust reproducibility of the proteomic approach employed, supporting its suitability for large-scale quantitative proteomic profiling. In-between the test and validation substudies, the proteomic data acquisition method was improved to double its throughput while maintaining high protein identification rates (shortening of LC gradient, adjusting SWATH window design to shorter gradient). Consequently, the validation cohort was analyzed using the enhanced data acquisition method. As no direct comparisons of proteomic profiles acquired in the test and the validation study were performed, the datasets were deemed suitable for this study setup. In this context, clustering analysis of the test study data showed that sample replicates from the same patient were the primary determinant of clustering, further emphasizing the reproducibility of DIA-MS measurements. Independent cohort replication on the same peptide-centric MS workflow isolates FT effects from biological heterogeneity and run-to-run technical variation, directly addressing whether observed patterns are robust in real-world discovery settings. The validation study revealed a distinct clustering trend between fresh and FT samples, indicating a global and systemic effect of the FT cycle on the proteome. Despite of the observed low effect sizes, 11 out of 30 initially identified target protein candidates were validated across both studies and their FT sensitivity should be considered in biomarker studies. In line, these validated proteins were involved in various biological processes, indicating that FT cycle sensitivity is not limited to specific functional protein groups.

Furthermore, the validated FT-sensitive proteins exhibited diverse physiochemical properties, including thermodynamically stable and unstable proteins. A majority were classified as unstable and hydrophobic. The predominance of hydrophobic proteins reflect heightened susceptibility to cold denaturation at ice-water interfaces, where reduced hydrophobic effect at subzero temperatures weaken protein core packing and promote partial unfolding.^17^ Indeed, the hydrophobic effect is recognized as a critical determinant in maintaining protein core stability during protein folding and cold denaturation.^18^^,^^19^^,^^20^^,^^21^ At lower temperatures, a reduction in the hydrophobic effect can lead to an increased tendency for cold denaturation of proteins.^22^ Therefore, hydrophobic proteins may be more susceptible to the effects of the FT cycle than their hydrophilic counterparts. Additionally, observed differential protein quantities after one FT-cycle likely result from forms of cryo-damage. Cryo-damage during freezing and thawing is mainly due to reversible protein aggregation caused by ice crystal growth,^23^^,^^24^^,^^25^^,^^26^ including cryoconcentration^27^—which generated localized pH shifts and elevated ionic strengths destabilizing thermodynamically unstable proteins^28^ - contact with interfaces,^29^^,^^30^ and cold denaturation.^31^^,^^32^ While reduced protein abundance typically indicates irreversible aggregation at ice-water interfaces and through cryoconcentration, increased quantities may result from degradation and misfolding of protein complexes. Though a significant alteration in protein detectability was identified for many proteins, a significant percentage of identified proteins displayed either no FT sensitivity following a single cycle in any dataset (28.2%) or exhibited inconsistent FT sensitivity across datasets (50%), and therefore, maintain their stability, at least after one FT cycle.

While orthogonal assays (e.g., immunoassays) represent the gold standard for confirming absolute protein concentration changes, such validation was beyond the scope of this pre-analytical characterization study. Future work should prioritize orthogonal confirmation for a subset of FT-sensitive proteins, particularly in the context of specific translational applications.

Another finding that stands out from the results pertains to the potential impact of sample storage conditions on biomarker detection in a cohort of 37 patients diagnosed with pancreatic disease (27 malignant, ten benign). LASSO regression detected 29 features as important in distinguishing between benign and malignant samples. Notably, two of these proteins, IGHV6-1 and CP, were also identified as FT-sensitive, highlighting the need to evaluate pre-analytical impact factors during the translation of biomarkers in the future. Both showed higher median log_2_ intensities in malignant samples, suggesting their potential as biomarkers for malignant pancreatic cancer. This biomarker discovery analysis served as a proof-of-concept demonstration, highlighting the potential influence of this pre-analytical factor on biomarker study outcomes.

This study highlights the importance of high-quality samples and standardized handling in biomarker research. Using DIA-MS, it demonstrates the significant impact of FT cycles on relative protein signal intensities in stored serum samples, by detecting proteomic changes after one FT cycle within the studies detection rate. This pre-analytical impact factor may explain why many proposed protein biomarkers fail to translate to clinical applications. The findings underscore the need for rigorous quality management in biospecimen handling and could improve biomarker research. Therefore, candidate biomarkers should be rigorously evaluated for their stability under clinically relevant conditions or for their compatibility with routine clinical sample handling, to ensure successful clinical translation.

### Limitations of the study

This study has several limitations that should be considered when interpreting the findings. First, the test and validation cohorts were not matched for disease type, stage, age, or sex, reflecting heterogeneity of real-world biobank material but also introducing potential confounding by biological variation. Second, the two cohorts were analyzed using slightly different DIA-MS acquisition settings after method optimization, which improved throughput but may have influenced direct comparability between datasets despite consistently high identification rates. Third, the study focused on relative protein abundances rather than absolute concentrations, and orthogonal validation by targeted assays was beyond the scope of this work. Finally, the analysis was restricted to serum proteins detectable by the applied DIA workflow, meaning that. Very low-abundance proteins or analytes outside the detection range of the method may be affected differently by FT exposure and were not captured here.

## Resource availability

### Lead contact

Requests for further information and resources should be directed to and will be fulfilled by the lead contact, Timo Gemoll (timo.gemoll@uni-luebeck.de).

### Materials availability

This study did not generate new unique reagents.

### Data and code availability

• Raw mass spectrometry data and search engine results have been deposited at the ProteomeXchange Consortium via the PRIDE partner repository with the dataset identifier PXD060473 and are publicly available as of the date of publication. De-identified patient data are available in the supplementary files.
• This study does not report original code.
• Any additional information required to reanalyze the data reported in this study is available from the lead contact upon request.

## Acknowledgments

This work was supported by the German Network for Bioinformatics Infrastructure-de.NBI, service center BioInfra.Prot, funded by the 10.13039/501100002347German Federal Ministry of Education and Research (BMBF)-Grant FKZ 031 A 534A. T. Sauer is grateful for the scholarship he received from the Ad Infinitum Foundation. Additionally, we thank Julia Horn, Katja Klempt-Gießing, and Emma Neumann for their excellent technical assistance in this project. The data have been published as a preprint on bioRxiv (BIORXIV/2025/647925). This study was performed as a part of the AKELOP project (project number LPW-E/1.2.2/673) supported by State Program Economy 2014–2020 with funds from the European Regional Development Fund (ERDF) and the state Schleswig-Holstein. Thorben Sauer received a doctoral degree scholarship from the Ad Infinitum foundation.

## Author contributions

T.S.: data curation, formal analysis, investigation, methodology, software, validation, visualization, and writing – original draft; M.O.: resources and writing – review and editing; R.M.: resources and writing – review and editing; H.R.: data curation and writing – review and editing; R.B.: resources and writing – review and editing; K.H.: resources and writing – review and editing; U.W.: resources and writing – review and editing; J.H.: resources and writing – review and editing; C.B.: resources and writing – review and editing; T.K.: resources and writing – review and editing; S.S.: methodology, data curation, and writing – review and editing; T.G.: funding acquisition, project administration, resources, validation, and writing – original draft. All authors read and approved the final manuscript.

## Declaration of interests

The authors declare no competing interests.

## Declaration of generative AI and AI-assisted technologies in the writing process

During the preparation of this work the authors used Perplexity AI in order to improve the language rigor. After using this tool/service, the authors reviewed and edited the content as needed and take full responsibility for the content of the published article.

## STAR★Methods

### Key resources table

**Table:** 

REAGENT or RESOURCE	SOURCE	IDENTIFIER
**Chemicals, peptides, and recombinant proteins**		

Acetone 100%	Merck	Cat#100014
Acetonitrile	Thermo Scientific	Cat#15578664
Ammonium bicarbonate	Honeywell	Cat#40867-50G
Dithiothreitol	BioRad Laboratories	Cat#1610610
Formic acid	Thermo Scientific	Cat#15508664
Iodoacetamide	BioRad Laboratories	Cat#1632109
Sodium deoxycholate	Sigma-Aldrich	Cat#302-95-4
Trypsin	Promega	Cat#V5280

**Deposited data**		

Proteomic dataset deposit	PRoteomics IDEntifications Database	https://www.ebi.ac.uk/pride/archive/projects/PXD060473

**Software and algorithms**		

DIA-NN (v1.8.1)	Demichev et al.^33^	https://github.com/vdemichev/DiaNN
DIA-NN R package (v1.0.1)	Demichev et al.^33^	https://github.com/vdemichev/diann-rpackage
R (v4.4.2)	R Foundation for Statistical Computing,^34^	https://www.R-project.org/
R-Studio	Posit, PBC	https://posit.co/download/rstudio-desktop/
DEP R package (v1.26.0)	Zhang et al.^35^	https://bioconductor.org/packages/DEP/
MSnbase R package	Gatto and Lilley,^36^; Gatto et al.^37^	https://bioconductor.org/packages/MSnbase/
crosstable R package (v0.8.1)	Chaltiel,^38^	https://CRAN.R-project.org/package=crosstable
ggpubr R package (v0.6.0)	Kassambara,^39^	https://CRAN.R-project.org/package=ggpubr
nlme R package (v3.1-167)	Pinheiro and Bates,^40^	https://CRAN.R-project.org/package=nlme
stats R package (base R, v4.4.2)	R Foundation for Statistical Computing,^34^	https://www.R-project.org/
corrplot R package (v0.95)	Simko and Wei,^41^	https://CRAN.R-project.org/package=corrplot
clusterProfiler R package (v4.14.6)	Yu et al.	https://bioconductor.org/packages/clusterProfiler/
Peptides R package	Osorio et al.^11^	https://CRAN.R-project.org/package=Peptides
tidymodels R package (v1.3.0)	Kuhn and Wickham,^42^	https://CRAN.R-project.org/package=tidymodels
tidyverse R package collection (v2.0.0)	Wickham et al.^43^	https://CRAN.R-project.org/package=tidyverse
dplyr R package (v1.1.4)	Wickham et al.^43^	https://CRAN.R-project.org/package=dplyr
ggplot2 R package (v3.5.1)	Wickham, 2016	https://CRAN.R-project.org/package=ggplot2
SWATH Variable Window Calculator V1.1	AB Sciex	https://sciex.com/
UniProtKB/Swiss-Prot database (v2020/12/6 and v2024/10/28)	UniProt Consortium,^44^	https://www.uniprot.org/
Pax.Db Human serum reference (v6.0)	Wang et al.^45^	https://pax-db.org/

### Experimental model and study participant details

The study concept and workflow are visualized in Figure 1A. Human serum samples utilized in this study were sourced from samples collected for routine proteomic profiling at the Section for Translational Surgical Oncology and Biobanking, Department of Surgery, University of Lübeck and University Hospital Schleswig-Holstein, Campus Lübeck, Germany. Samples were obtained from adult patients prior to surgical intervention, during routine check-up appointments, or at follow-up visits. No exclusion criteria were applied to sample selection for this study. Comprehensive patient characteristics are detailed in Tables 1 and S1. Patients were categorized as either benign or malignant (‘category’ variable) based on their diagnosed condition. Patients whose blood samples were collected during post-surgical follow-up appointments were classified as ‘follow-up’ in the category and the ‘sampling’ variable. The ‘sampling’ variable further specifies the context of blood sample acquisition: ‘surgery’ denotes samples collected immediately before tumor removal surgeries, while ‘routine’ indicates samples collected during routine monitoring consultations within the Department of Surgery (without surgery).

The test study cohort consisted of three to four serum samples from 25 patients (acquired simultaneously), including 18 patients diagnosed with tumors (pancreatic, colorectal, and lung) and seven patients with non-neoplastic conditions. A total of 99 fresh and frozen-thawed (FT) serum sample pairs were analyzed in the test study. The median storage duration for the FT samples was 3.6 months (Inter-quartile-range 2.8; 7.1, standard deviation 2.5).

The validation study utilized single measurements of 109 fresh and FT serum sample pairs. This cohort included serum samples from 94 patients with malignant pancreatic and colorectal cancers, as well as 15 patients with non-malignant pancreatic and colorectal conditions. The median storage duration of the FT samples within the validation cohort was 9.1 months (Inter-quartile-range 6.7; 19.9, standard deviation 11.0). The test and validation cohort were compared using the R *crosstable* package (v0.8.1).^38^

The research protocols were approved by the local ethics committees of the University of Lübeck (#16–281, #16–282, #17–043, and #19-147A, #2023-362). All participating patients provided informed written consent.

### Method details

#### Serum sample acquisition

Following the acquisition of signed informed consent, peripheral blood was collected by the standard operating procedures established at the hospital-based Interdisciplinary Center for Biobanking Lübeck (University Hospital Schleswig-Holstein, Campus Lübeck, Germany). Patient blood samples were collected in 9 mL S-Monovettes Serum Gel (Sarstedt, Germany), allowed to cool at room temperature for 30 min, and subsequently centrifuged at 1,500 × g, 10 min and 4°C. The serum was then aliquoted into 0.7 mL cryovials (Azenta Life Sciences, USA). Before freezing in liquid nitrogen, a 10 μL sub-aliquot of serum was transferred to 1.5 mL low protein binding tubes and stored on ice during transport to the Section of Translational Surgical Oncology and Biobanking laboratories for immediate sample preparation and protein digestion.

#### Protein digestion

For FT sample preparation, stored serum samples were retrieved from liquid nitrogen. Serum samples were thawed at room temperature until fully soluble. A 2 μL aliquot of serum was combined with 18 μL Milli-Q/ultra-pure (MQ) water. Subsequently, 10 μL of the diluted serum was added to 40 μL of ice-cold 100% acetone (Merck, USA), vortexed, and incubated at −20°C for 1h. The serum was then centrifuged for 10 min at 10,000 × g and 4°C. The supernatant was carefully removed and discarded, while the resulting pellet was allowed to air-dry. The pellet was resuspended in 100 μL deoxycholate (DOC) buffer (7.9 mg ammonium bicarbonate (Honeywell, USA), 1 mL Milli-Q water, and 10 mg sodium deoxycholate (Sigma-Aldrich, USA)) and 1 μL 1,4-dithiothreitol 1M (BioRad Laboratories, USA) solution in DOC buffer. This solution was incubated at 60°C for 30 min. Subsequently, 4 μL of 0.5M iodoacetamide (BioRad Laboratories, USA) was added and incubated for 30 min in the dark. Afterward, 1 μL of trypsin (Promega, Germany) was added, and the mixture was incubated overnight at 37°C. The digestion was quenched with the addition of 1 μL of formic acid (Thermo Scientific, USA), and the samples were centrifuged for 5 min at 14,000 × g. The resulting supernatant was transferred to a new tube and frozen at −80°C. Thereafter, the samples were lyophilized for 3h in a vacuum centrifuge (Christ Gefriertrocknungsanlagen GmbH, Germany). The serum digest was then stored at −20°C until further use.

#### High-performance liquid chromatography

The serum sample digests were solubilized in 50 μL loading buffer and separated using a Dionex Ultimate 3000 high-performance liquid chromatography (HPLC) system (Thermo Fisher, USA). The HPLC was configured with a precolumn (μ-Precolumn Acclaim PepMap100, 0.3 mm × 5 mm, 5 μm particle size, 100 Å pore size, Thermo Fisher Scientific, USA). A Luna C18(2) (0.3 mm × 50 mm, 3 μm particle size, 100 Å pore size, Phenomenex Inc., USA) was used as an analytical column. Although the identical chromatographic system was employed for both study cohorts, the gradient settings differed between the test and validation studies. A 90-min gradient was employed to analyze the test study cohort serum samples. The method commenced with a 4 min sample injection and trap column loading, utilizing an initial solvent composition of 97% solvent A (1% formic acid in MQ water) and 3% solvent B (1% formic acid in acetonitrile (Thermo Scientific, USA)). Subsequently, a linear gradient was applied, increasing the proportion of solvent B to 25% at 68 min, 35% at 73 min, and finally reaching 80% at 80 min, which was sustained for 3 min. Subsequently, the concentration of solvent B was gradually reduced to 3% over a 4 min period, which was maintained for 8 min.

The validation study employed a 45-min gradient, proportionally shortened to the 90-min gradient used in the test study, to increase the sample throughput. Accordingly, the 45 min gradient profile comprised a 4 min sample injection and trap column loading at 97% solvent B to 3% solvent A, followed by a linear gradient, increasing the proportion of solvent B to 25% at 30.68 min, 35% at 32.94 min, and 80% at 33.84 min until 35.2 min. Subsequently, the proportion of solvent B was reduced to 3% at 37 min and maintained for 8 min. A flow rate of 5 μL/min was used for all HPLC methods.

#### Mass spectrometry

Peptides eluting from the Dionex Ultimate 3000 HPLC were analyzed using a TripleTOF 5600+ (AB Sciex, USA) mass spectrometer system. For serum sample data acquisition, data-independent acquisition-MS in sequential window acquisition of all theoretical mass spectra (SWATH) was employed. The following acquisition parameters were used: Ion Spray Voltage Floating at 5000 V; ion source gas (GS1), 15; ion source gas (GS2), 0; curtain gas at 30 and source temperature heating set to 0 °C. The optimized declustering potential was set at 100; collision energy to 19.2; collision energy spread, 5.0; ion release delay, 67; ion release width at 25. One 0.05 s MS scan (m/z 350–1250) was performed for data acquisition. Based on the 90- and 45-min HPLC gradients, two 100 precursor isolation window SWATH methods were developed. The precursor isolation windows were defined using the SWATH Variable Window Calculator V1.1 (AB Sciex, USA) based on precursor *m*/*z* densities obtained from data-dependent acquisition (DDA) spectra. The mass range was set to 350–1250 m/z, window overlap was set to 1.0 Da, and collision energy spread was fixed at 5.0 eV. For DDA acquisition, identical instrument working parameters were used. MS scans were performed for 350–1250 m/z with an accumulation time of 0.25 s, and MS/MS scans were performed for 100–1500 m/z with an accumulation time of 0.05 s at high sensitivity mode.

### Quantification and statistical analysis

#### General statistical reporting

All statistical tests, the exact value of n, the biological unit represented by n, the definition of center, and the dispersion and precision measures applied in each analysis are specified in the corresponding figure legends (Figures 1, 2, 3, and 4; Figure S1) and in the Results section (subsections “Study workflow and cohort description”, “Proteomics data quality and comparability”, “FT cycle as pre-analytical impact factor”, and “FT-sensitive proteins are potential biomarker candidates”). Detailed per-protein statistical results are provided in Tables S2, S3, S4, S5 and S6 and in Figure S1.

Throughout the manuscript, *n* refers to the number of fresh/freeze–thawed (FT) serum sample pairs analyzed by DIA-MS, with each pair derived from one individual patient. The test cohort comprised *n* = 99 fresh–FT pairs from 25 patients (3–4 paired aliquots per donor, acquired simultaneously), and the validation cohort comprised *n* = 109 fresh–FT pairs from 109 patients (one pair per donor); patient- and sample-level characteristics are summarized in Tables 1 and S1, and visualized in Figures 1B and 1C. For the biomarker discovery sub-analysis (Figure 4), *n* = 37 fresh serum samples from 37 validation-cohort patients were used (27 PDAC, 10 benign pancreatic lesions).

Continuous demographic and sample variables are reported as median [first quartile; third quartile] and mean (SD) (Table 1). Boxplots show the median (center line), first-to-third quartile (box), and 1.5×IQR whiskers; violin plots additionally display quartile lines (Figures 2C and 2F). Heatmap values in Figure 3G correspond to Z-score-transformed mean protein intensities calculated per cohort and condition. Unless otherwise stated, all tests were two-sided and statistical significance was defined as *p* ≤ 0.05 after Benjamini–Hochberg (BH) multiple-testing correction where applicable. Asterisk conventions are defined in each figure legend and follow: ∗*p* ≤ 0.05, ∗∗*p* ≤ 0.01, ∗∗∗*p* ≤ 0.001.

#### SWATH data processing

The raw SWATH data from both study sub-cohorts were processed using the software tool *DIA-NN* v1.8.1 with identical settings.^33^ The software was used in the high-accuracy LC mode with retention time-dependent cross-normalization enabled. Mass accuracy was fixed to 2e−05 (MS2) and 1.5e−05 (MS1), while scan window width was determined automatically. The ‘match between runs’ function was used to develop a spectral library using the ‘smart profiling strategy’ from the data-independent acquisition data. The human UniProtKB/swiss-prot database (version 2020/12/6)^44^ was used for protein inference from identified peptides. Trypsin/P was specified as protease. The precursor ion generation settings were set to a peptide length of 7–52 amino acids, the maximum number of missed cleavages to one. The maximum number of variable modifications was set to zero. N-terminal methionine excision and cysteine carbamidomethylation were enabled as fixed modifications.

#### Statistical analysis and bioinformatics

##### Protein quantity data preprocessing

Descriptive statistics and comparisons between the test and validation cohorts (Table 1; Figures 1B and 1C) were computed using the R *crosstable* package (v0.8.1).^38^ Between-cohort differences in continuous variables (patient age, storage duration) were assessed using the two-sided Wilcoxon rank-sum test; asterisks in Figure 1B denote ∗∗∗*p* ≤ 0.001 (see Figure 1 legend). Categorical variables (sex, organ, diagnosis, sample category, sampling context) are reported as absolute counts and percentages (Table 1).

##### Protein quantity data preprocessing

The DIA-NN report files were further processed in the *DIA-NN R* package (v1.0.1)^33^ for MaxLFQ-based protein quantification.^46^ For the test and validation dataset, a report was generated containing the unique proteins that passed the FDR cut-off of 0.01 applied at the precursor level and were identified and quantified using proteotypic peptides only. A minimum of two unique peptides identified all proteins included in the final datasets. The proteins were mapped to their corresponding gene names; thus, the terms ‘proteins’ and ‘genes’ are used interchangeably in this study. The quantitative data were further pre-processed using the R package *DEP* (v1.26.0).^35^ The unique protein datasets were filtered for data completeness: each protein had to be identified in at least 70% of samples. A variance stabilizing normalization^47^ was applied to the data, incorporating a log_2_ transformation, prior to imputing the residual missing values using the k-nearest neighbor model, as no missing not at random patterns were identified. Missing value rates were 2.69% for the test dataset, and 2.13% for the validation dataset. DEP borrows the imputation function from *MSnbase* and was used with default settings.^36^^,^^37^

##### Proteomic data quality assessment

The two acquired proteomic datasets were assessed on their reproducibility across the acquisition periods by evaluating the protein and peptide identifications, normalized intensity ranges, and coefficients of variation (CV) across acquisition batches (test cohort: 5 batches, *n* = 198 samples across 43 weeks; validation cohort: 6 batches, *n* = 218 samples across 60 weeks; Figures 2A–2F). Differences within CV between batches was assessed utilizing a one-way ANOVA (fixed factor: batch) and/or pairwise two-sided Student’s *t* test, corrected for multiple testing with the Benjamini-Hochberg procedure,^48^ using the *ggpubr* package (v0.6.0).^39^ CV distributions are visualized as violin plots with quartile lines (Figures 2C and 2F); ANOVA *p*-values and the significance threshold for pairwise Student’s t-tests (∗, adj.*p* ≤ 0.05) are reported in the Figure 2 legend and in the Results (“Proteomics data quality and comparability”). The number of proteins identified per sample and distribution of identified peptides per protein (mean and SD reported in the in-panel tables of Figures 2B and 2E) are summarized in Figures 2A, 2B, 2D, and 2E.

##### Assessment of FT cycle impact

A linear mixed model (LMM) was used to perform a two-group comparison of paired fresh and FT sera for each protein separately, employing the *nlme* R-package (v3.1-167),^40^ with *n* = 99 paired fresh/FT observations from 25 patient in the test cohort and *n* = 109 paired observations from 109 patients in the validation cohort. Protein abundance was modeled as dependent variable with type of sample (fresh or FT) as fixed and the subject ID as random effects (the latter accounting for the repeated-measures design of the test cohort). The Benjamini-Hochberg procedure was used to adjust *p*-values for sample type.^48^ A significant difference in protein levels between fresh and FT samples was considered at q < 0.05. Per-protein log_2_ fold changes, raw *p*-values, and BH-adjusted q-values are provided in Table S2 (test cohort, related to Figure 3B) and Table S3 (validation cohort, related to Figure 3B) and are visualized as volcano plots in Figure 3B. Cross-cohort overlap of differentially abundant proteins is displayed in Figure 3C (Venn diagram) and Figure 3D (scatterplot of cohort-matched log_2_ fold changes); the 11 validated FT-sensitive proteins are listed with stability status and Pax.Db abundance class in Table S4 and ranked in Figure 3E.

Principal component analyses (PCA) were calculated for both study cohorts using the base R *stats* package (v4.4.2)^34^ on the pre-processed, log_2_-transformed proteins intensity matrices. Percent variance explained per component is indicated in parentheses in the axis labels of Figure 3A; adjacent density curves show the kernel density distributions of fresh and FT samples along PC1 and PC2.

Correlation analysis was carried out using the *ggpubr* package (v0.6.0)^39^ and the R package *corrplot (v0*.*95)*^41^ utilizing the protein intensity data of the validation cohort. Per protein Pearson correlation coefficient (R) and the corresponding two-sided *p*-values were calculated between log_2_ protein intensity and storage duration in liquid nitrogen (*n* = 109 FT samples from 109 patients), and between storage duration and patient age. The full correlation results are visualized in Figure S1 and summarized in the Results (“FT cycle as pre-analytical impact factor”), where the highest correlation coefficients are reported.

he proteome abundance database (Pax.Db, v.6.0) Human serum reference list was utilized to retrieve typical serum protein concentrations.^45^ The Pax.Db reference list was then categorized based on protein concentrations, resulting into three distinct abundance classes: high abundant proteins (top 25%), mid-abundant proteins (mid 50%), and low-abundant proteins (low 25%). The categorized reference list was the mapped to the proteomic dataset under investigation for the purpose of abundance stratification (Figures 3E; Table S4). The R package clusterProfiler (v4.14.6) function groupGO was used for acquiring functional profiles for the target proteins at gene ontology term level three (biological process subontology). The top10 enriched GO:BP terms, ranked by gene ratio, are visualized as a chord plot in Figure 3F, complete mapping results are provided in Table S5.

The *tidyverse* package (v2.0.0) collection was employed throughout all bioinformatics analyses to facilitate data handling and visualization.^43^ Key packages used include *dplyr (v1*.*1*.*4)* for data manipulation and *ggplot2 (v3*.*5*.*1)* for visualization.

##### Physiochemical property mapping

For validated target proteins (*n* = 11 proteins validated as FT-sensitive in both cohorts), the physiochemical properties hydrophobicity index (Kyte & Doolittle scale),^49^ the instability index,^50^ and the aliphatic index^51^ were calculated using the Peptides R package developed by Osorio et al.^11^ The complete amino acid sequences of the proteins were extracted from UniProtKB/swiss-prot database (version 2024/10/28) and used to calculate the parameters. The variables hydrophobic index, instability index, and aliphatic index were discretized for better visualization as follows: hydrophobicity: >0 ≙ hydrophobic, <0 ≙ hydrophilic; instability index: >40 ≙ unstable, <40 ≙ stable; aliphatic index: <70 ≙ low, 70–90 ≙ moderate, >90 ≙ high. Discretized values are displayed as heatmap annotations in Figure 3G; continuous values and the underlying amino acid sequences are reported in Table S6.

##### Identification of biomarker candidates using machine learning

Fresh serum proteomics from a sub-cohort of 37 patients within the validation cohort, encompassing individuals with pancreatic ductal adenocarcinoma (PDAC) (*n* = 27) and benign lesions (*n* = 10), were analyzed to identify potential biomarkers. Preprocessing included the removal of zero-variance predictors and the z-transformation of all predictor variables. A dataset split was applied to the data, with 80% of the data allocated to the training and 20% to the testing set. Stratification was performed on the target variable (PDAC vs. Benign).

A Least Absolute Shrinkage and Selection Operator (LASSO) regression model was employed to discern key features for classifying samples into benign and malignant categories using the *tidymodels (v1*.*3*.*0)* framework.^42^ The penalty hyperparameter was tuned by 10-fold cross-validation across a grid of penalty values ranging between 1e^−10^ and 1e^10^. To enhance feature selection, the 10^th^ best lambda value was selected based on the root mean squared error RMSE. This less stringent approach permitted the inclusion of additional features in the final model. The final LASSO model was then fitted to the training data, and non-zero coefficients (feature importances) were extracted to identify the selected features and their impact on the target variable in terms of classification in the malignant category. Coefficients are reported in the Results (“FT-sensitive proteins are potential biomarker candidates”, Figure 4A). Group level comparison of log_2_ intensities of the two FT-sensitive candidates CP and IGHV6-1 between malignant (*n* = 27 PDAC) and benign (*n* = 10) fresh serum samples was performed using a two-sided Student’s *t* test; exact *p*-values are reported in Figure 4B.

#### Software and reproducibility

All downstream statistical analyses and visualizations were performed in R (v4.4.2; R Foundation for Statistical Computing (44)). Key packages included *crosstable* (v0.8.1) (33), *DIA-NN* R (v1.0.1) (34), *DEP* (v1.26.0) (37), *MSnbase* (39,40), *nlme* (v3.1-167) (43), *ggpubr* (v0.6.0) (42), *corrplot* (v0.95) (45), *clusterProfiler* (v4.14.6), *Peptides* (11), *tidymodels* (v1.3.0) (51), and the *tidyverse* collection (v2.0.0) (47). Raw SWATH-MS data were processed in *DIA-NN* (v1.8.1) (34).

#### Data availability

The mass spectrometry data have been deposited at the ProteomeXchange Consortium via the PRIDE partner^52^ repository with the dataset identifier PXD060473.

## Supplemental information

Document S1. Figure S1

Table S1. Patient cohort clinical and meta data

Table S2. Differential expression analysis results of the test cohort study, related to Figure 3B

Table S3. Differential expression analysis results of the validation cohort study, related to Figure 3B

Table S4. Protein stability status and abundance according to serum Pax.Db, related to Figure 3E

Table S5. Gene ontology “biological process” mapping results of the validated target proteins, related to Figure 3F

Table S6. Physiochemical annotation data of the validated target proteins, related to Figure 3G
