# Supplementary material for: Impact of single freeze-thaw cycles on human serum proteins: Implications for mass spectrometry biomarker validation
Source: iScience. 2026 Apr 28;29(6):115934. doi: 10.1016/j.isci.2026.115934 (PMC13206734; doi:10.1016/j.isci.2026.115934)
Supplement: Document S1. Figure S1 [file mmc1.pdf]

iScience, Volume *29*

Supplemental information

Impact of single freeze-thaw cycles on human serum proteins: Implications for mass spectrometry biomarker validation

Thorben Sauer, Martina Oberländer, Regina Maushagen, Helene Radloff, Ruediger Braun, Kim Honselmann, Ulrich Wellner, Jens Habermann, Claudia Benecke, Tobias Keck, Silke Szymczak, and Timo Gemoll

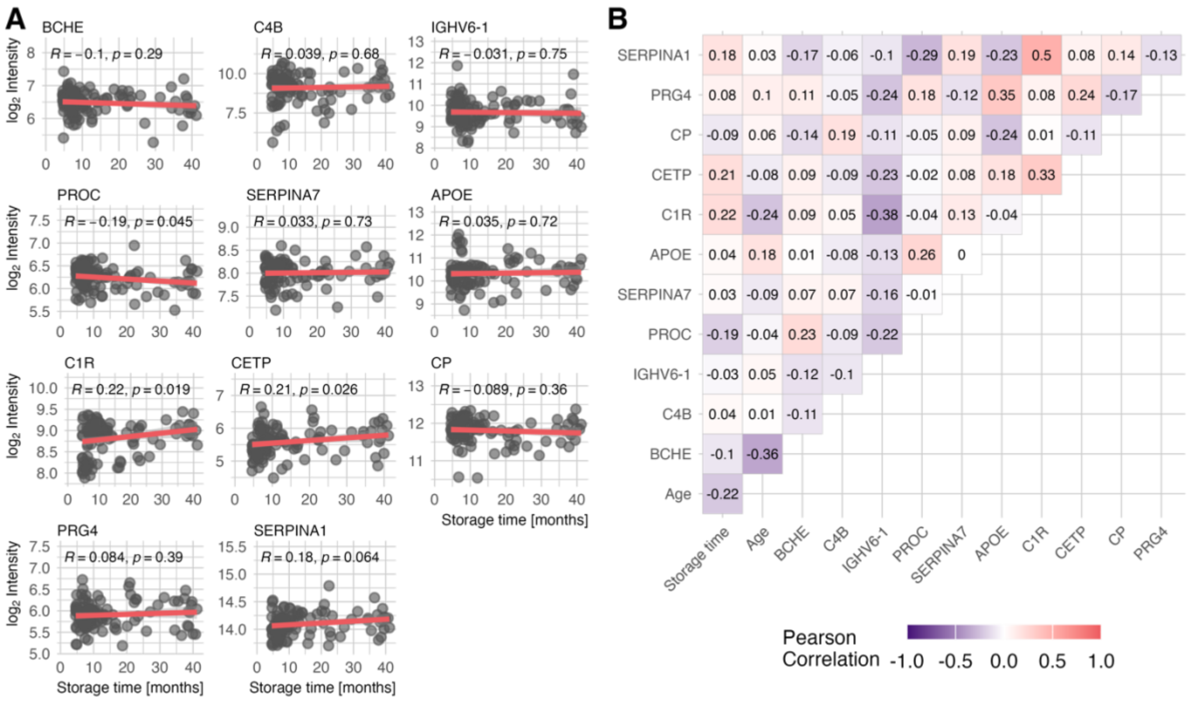

Supplementary Figure 1: Correlation between protein abundance and storage time in FT samples. ***A:*** *Scatter plots of* $\log_2$ *protein intensity and liquid nitrogen storage time in months with linear model trendline and Pearson correlation coefficient. No meaningful correlation was found between protein abundance and storage time of the serum samples.* ***B:*** *No correlation was found between the target proteins and storage time or patient age. Age was included as a negative control variable.*
